# Applications of machine learning in pine nuts classification

**DOI:** 10.1038/s41598-022-12754-9

**Published:** 2022-05-25

**Authors:** Biaosheng Huang, Jiang Liu, Junying Jiao, Jing Lu, Danjv Lv, Jiawei Mao, Youjie Zhao, Yan Zhang

**Affiliations:** 1 College of Big Data and Intelligent Engineering, Southwest Forestry University, Kunming, 650224 Yunnan China; 2 Key Laboratory for Forest Resources Conservation and Utilization in the Southwest Mountains of China, Ministry of Education, Southwest Forestry University, Kunming, 650224 Yunnan China; 3 College of Forestry, Southwest Forestry University, Kunming, 650224 Yunnan China; 4 College of Mathematics and Physics, Southwest Forestry University, Kunming, 650224 Yunnan China

**Keywords:** Computer science, Machine learning, Classification and taxonomy

## Abstract

Pine nuts are not only the important agent of pine reproduction and afforestation, but also the commonly consumed nut with high nutritive values. However, it is difficult to distinguish among pine nuts due to the morphological similarity among species. Therefore, it is important to improve the quality of pine nuts and solve the adulteration problem quickly and non-destructively. In this study, seven pine nuts (*Pinus bungeana*, *Pinus yunnanensis*, *Pinus thunbergii*, *Pinus armandii*, *Pinus massoniana*, *Pinus elliottii* and *Pinus taiwanensis*) were used as study species. 210 near-infrared (NIR) spectra were collected from the seven species of pine nuts, five machine learning methods (Decision Tree (DT), Random Forest (RF), Multilayer Perceptron (MLP), Support Vector Machine (SVM) and Naive Bayes (NB)) were used to identify species of pine nuts. 303 images were used to collect morphological data to construct a classification model based on five convolutional neural network (CNN) models (VGG16, VGG19, Xception, InceptionV3 and ResNet50). The experimental results of NIR spectroscopy show the best classification model is MLP and the accuracy is closed to 0.99. Another experimental result of images shows the best classification model is InceptionV3 and the accuracy is closed to 0.964. Four important range of wavebands, 951–957 nm, 1,147–1,154 nm, 1,907–1,927 nm, 2,227–2,254 nm, were found to be highly related to the classification of pine nuts. This study shows that machine learning is effective for the classification of pine nuts, providing solutions and scientific methods for rapid, non-destructive and accurate classification of different species of pine nuts.

## Introduction

There are more than 113 formally recognized species of *Pinus Linn* mainly distributed in the northern hemisphere^1, 2^, they form an important part of forest ecosystems. Pine nuts are the seeds of pine trees, they are a commonly consumed nut, and an important agent of afforestation and reproduction^3^. Pine nuts are rich in protein, fatty acids, minerals and vitamins. They also contain oleic acid, linolenic acid and other unsaturated fatty acids, which facilitate the prevention of cardiovascular disease^4^. Species recognition of pine nuts is important for food safety and pine nut quality. In recent years, the rising price of pine nuts has brought huge economic benefits. The global output of pine nuts in 2020–2021 is about 381,700 tons. China is the main import and export country of pine nuts in the world. Considering the visual similarity between pine nuts, the possibility of adulteration of products is very high, and the adulteration problem has a great impact on health and economy. Therefore, how to detect adulterated products in pine nuts in a convenient, fast and non-destructive way is a challenge to the food safety of pine nuts.

Presently, common methods of species identification include morphological analysis^5^, molecular marker technology^6–9^, protein electrophoresis^10^, liquid chromatography^11^, spectral analysis^12–14^ and image recognition^15^. Morphological analysis requires a high level of expertise that is not easily acquired and as such, due to large morphological similarity between some species, the rate of accurate identification is low^16^. Although the use of molecular markers returns a higher recognition rate and more accuracy, it is a destructive methodology, time-consuming, and limited by the number of published markers in the public databases. Therefore, this study establishes machine learning models for pine nut classification based on near-infrared (NIR) spectroscopy and images.

NIR spectroscopy is a methodology that makes use of molecular vibrations in the infrared spectrum in the material. The process of NIR spectroscopy involves the NIR apparatus emitting an infrared light that enters the sample. Here it is reflected, refracted, diffused and absorbed and finally carries the sample information back into the detector. This methodology is convenient, rapid, non-destructive, and cost-effective. It has been used in many agricultural fields, including research into wheat^17^, soybean^18^, cowpea^19^ and rice^12^ production. So far, there are few reports on the application of NIR spectroscopy in forestry and pine nut research. Specifically, Tigabu et al.^20^ collected visible-NIR spectral data of *Pinus sylvestris* nuts in different areas and preprocessed the spectral data by means of Multiplicative Scatter Correction (MSC). The nuts source was constructed through Soft Independent Modelling of Class Analogy (SIMCA) and Partial Least Squares Discriminant Analysis (PLS-DA). Loewe et al.^21^ collected NIR spectral data of Mediterranean *Pinus pinea* from Chilean plantations for classification. Moscetti et al.^22^ collected the NIR spectral data of the nuts of *P. pinea* and *Pinus sibirica* in different regions and established a spectral classification model by using PLS-DA and Interval PLS-DA (IPLS-DA) methods. However, the effects of other different classification models still need to be further discussed in more species of pine nuts.

Machine learning based on image has been successfully applied to rice pest identification^23^, *Dendrolimus punctatus* Walker damage detection^24^ and other agricultural and forestry fields. Deep learning, a type of machine learning, uses hierarchical analysis and multilevel calculation to obtain results. Deep convolutional neural network (CNN) has been successfully applied in image recognition for applications such as tomato pesto recognition^25^, fish image recognition^26^. Moscetti et al.^22^ collected the image data of the nuts of *P. pinea* and *P. sibirica* in different regions, carried out feature extraction, obtained 10 features based on image data, and used these features to construct image-based classification model. Although the feasibility of pine nuts classification has been proved based on manually extracted image-features, the automatic classification model is still worthy of further research in more species of pine nuts.

Therefore, the use of modern computer technology to classify pine nuts greatly promotes the research of non-destructive, rapid and accurate classification of pine nuts. In this study, machine learning technology is adopted, and the application potential of machine learning in pine nut classification is verified. The contributions of the current work are: (1) Molecular markers were used to identify pine nuts species; (2) NIR spectroscopy and images of 7 pine nuts (two kinds of edible pine nuts (*Pinus bungeana* and *Pinus armandii*) and five common species (*Pinus yunnanensis*, *Pinus thunbergii*, *Pinus massoniana*, *Pinus elliottii* and *Pinus taiwanensis*)) were collected. (3) NIR spectroscopy uses five machine learning methods for classification, while image recognition chooses five CNN models. This study verifies the potential of machine learning in pine nuts classification and provides a practical method for faster, non-destructive and accurate identification of pine nut species.

## Results

### Molecular markers

The assembled ITS2 and rbcL sequences were used to molecular markers by comparing to the GenBank database (https://www.ncbi.nlm.nih.gov/search/all/?term=blast). Table 1 shows that the ITS2 sequence length ranges from 477–482 bp while the rbcL gene length ranges from 677–720 bp **(**Table 2). The GenBank accession numbers are OK274058-OK274066 and OK271114-OK271122. The results show that *P. massoniana*, *P. armandii*, *P. thunbergii* and *P. bungeana* were recognized while *P. taiwanensis* (Synonyms is *Pinus hwangshanensis*) was not recognized. There were not the same species in GenBank compared with the ITS2 gene sequences of *P. yunnanensis* and *P. elliottii*. It is evident that ITS2 and rbcL are the suitable molecular markers for the species recognition of some pine nuts and molecular analyses are limited by data publicly available in GenBank. Then by consulting Kunming Institute of Botany, Chinese Academy of Sciences, the labels were carried out again to confirm the reliability and authenticity of pine nut species.

**Table 1:** ITS2 sequence markers results.

Sample_name	Gene length (bp)	GenBank species name	Query_Cover^a^ (%)	Per_Ident^b^ (%)	Accession number^c^
*P. massoniana*	479	*P. massoniana*	98	100	MH444832.1
*P. yunnanensis*	481	NA^d^	NA	NA	NA
		*P. thunbergii*	97	100	MH444826.1
*P. elliottii*	478	NA	NA	NA	NA
*P. armandii*	479	*P. armandii*	97	100	MH444830.1
*P.taiwanensis*	477	*P. taiwanensis*	84	100	JF829701.1
		*P. thunbergii*	98	100	MH444826.1
*P. thunbergii*	480	*P. thunbergii*	97	100	MH444826.1
*P. bungeana*	482	*P. bungeana*	79	100	MH703244.1

**Table 2:** rbcL sequence markers results.

Sample_name	Gene length (bp)	GenBank species name	Query_Cover^a^ (%)	Per_Ident^b^ (%)	Accession number^c^
*P. massoniana*	698	*P. massoniana*	100	100	MF564195.1
*P. yunnanensis*	677	*P. yunnanensis*	100	100	MK135067.1
		*P. thunbergia*	100	100	MH612862.1
*P. elliottii*	703	*P. elliottii*	100	100	NC_042788.1
		*Pinus teocote*	100	100	NC_039586.1
		*Pinus taeda*	100	100	KC427273.1
*P. armandii*	705	*P. armandii*	100	99.86	KP412541.1
*P.taiwanensis*	701	*P.hwangshanensis*	100	100	JN854194.1
		*P. thunbergii*	100	100	MH612862.1
*P. thunbergii*	704	*P. thunbergii*	100	100	JQ512594.1
*P. bungeana*	720	*P. bungeana*	100	100	MH612857.1

^a^Query_cover, the percentage of the sample sequence covered by the GenBank sequence.

^b^Per_Ident, the percentage similarity of the sample and GenBank sequences.

^c^Accession number, the GenBank accession number.

^d^NA, no match for the same species.

### Classification model based on NIR spectral data

**Figure 1:**
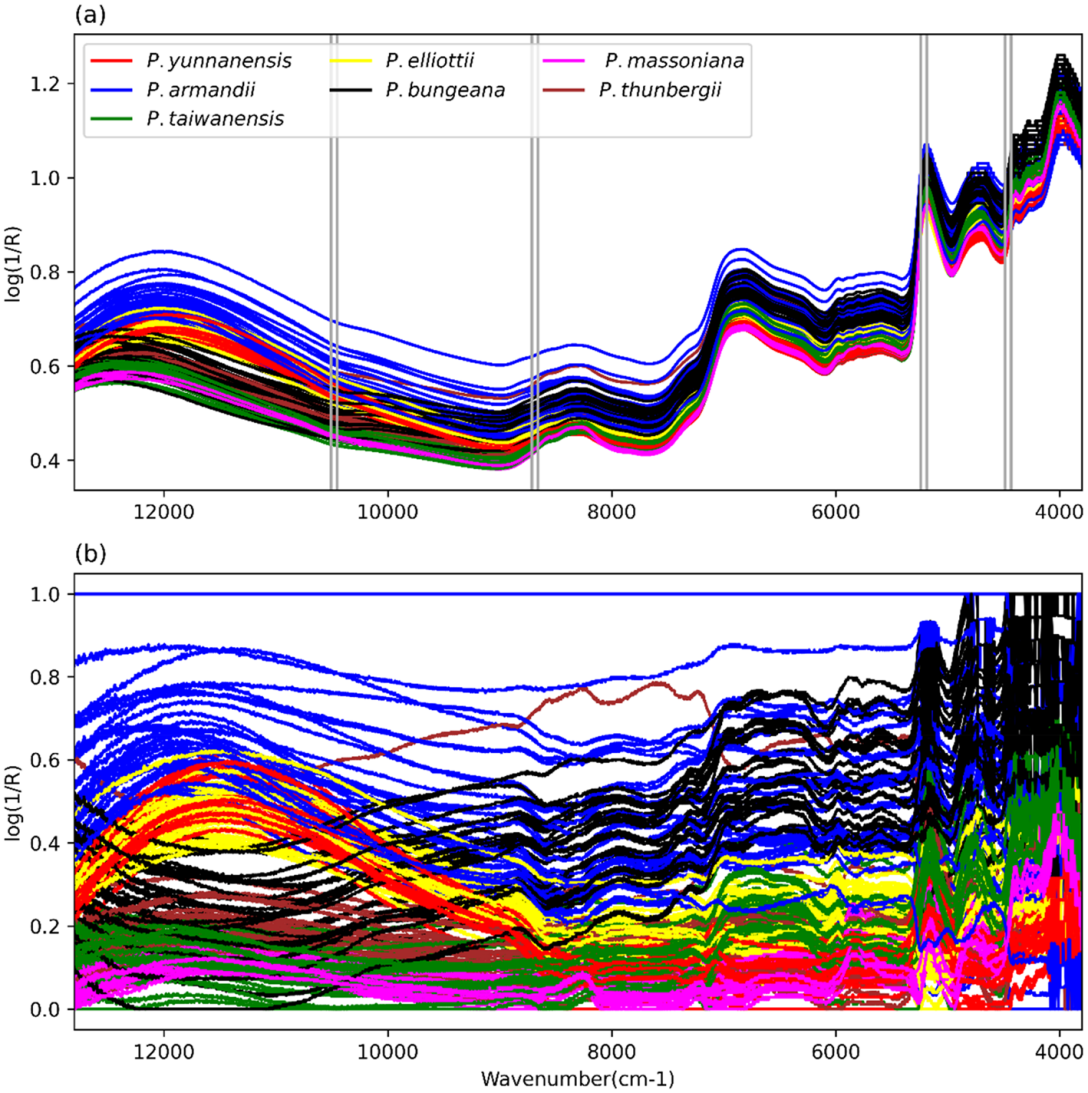
Pine nut NIR spectral data. (**a**) All of the original NIR spectra; (**b**) The normalized NIR spectra, R stands for reflectivity and log(1/R) represents absorbance. Vertical straight stripes represent the sensitive bands at 10,506.29–10,452.29 cm^−1^, 8712.813–8658.815 cm^−1^, 5241.572–5187.575 cm^−1^ and 4489.471–4435.474 cm^−1^ (951–957 nm, 1,147–1,154 nm, 1,907–1,927 nm, 2,227–2,254 nm) selected by moving sliding windows.

The collected pine nut NIR spectra were analyzed and are represented in Fig. 1. It is apparent from all original NIR spectra (Fig. 1a) that the amplitude, peaks and troughs of the NIR spectra of the seven pine nuts have similar changes. Among them, the value of *P. armandii* is at a higher position (indicating the highest absorbance value) compared to the whole range, and the value of *P. massoniana* is at a lower position. The normalized NIR spectra (Fig. 1b) show that the NIR spectrum of each pine nut is more distinct after normalization, and the changes between the pine nut values can be observed more clearly. Among them, *P. armandii* and *P. bungeana* are highly mixed in the range of 9,000–4,000 cm^−1^ (1,111–2,500 nm).

Ten independent analyses were carried out on normalized and non-normalized NIR spectral data using the five traditional machine learning models i.e., the Decision Tree (DT), Random Forest (RF), Multilayer Perceptron (MLP), Support Vector Machine (SVM) and Naive Bayes (NB) (Table 3). It is evident from Table 3 that the classification of pine nuts is effective using these models. When the data are not normalized, the accuracy of the DT and RF classification models is greater than 0.83. For normalized data, the classification accuracy of the five models is > 0.80, with MLP and SVM providing an accuracy of > 0.93. With pre-process of data, the performance of the MLP and SVM models have been greatly improved, the accuracy of the MLP model reaches 0.99, while the SVM model reaches 0.94. Overall, these results show that the RF model is a better classification method when the data are not normalized, while the MLP model is the best for normalized data.

**Table 3:** Classification model results based on full spectrum data.

Type	Model	Acc_max	Acc_min	Acc_avg
Non-normalized	DT	0.90	0.74	0.84
	MLP	0.71	0.36	0.54
	RF	0.95	0.79	0.86
	SVM	0.55	0.36	0.46
	NB	0.86	0.67	0.76
Normalized	DT	0.90	0.81	0.87
	MLP	1.00	0.98	0.99
	RF	0.90	0.86	0.89
	SVM	0.95	0.88	0.94
	NB	0.86	0.69	0.80

The precision (Pre) and F1-score (F1) are presented in Table 4 (non-normalized data) and Table 5 (normalized data). In Table 4, the precision and F1-score of *P. armandii* and *P. bungeana* are higher, and the precision of *P. bungeana* is the highest, reaching 0.97. However, the precision and F1-score of *P. taiwanensis* and *P. massoniana* are quite low reaching precision scores of 18% and 22% respectively. In Fig. 1a, the distinction between *P. armandii* and *P. bungeana* is clear, while the *P. taiwanensis* and *P. massoniana* are less distinct and thus more difficult to classify. However, Table 5 shows that the precision and F1-scores of the seven pine nut species are greatly improved after normalization. This indicates that data normalization is a necessary step for spectral data processing.

**Table 4:** Precision and F1 scores of five pine nut classification models with non-normalized NIR spectral data.

Number	Type	DT		MLP		RF		SVM		NB	
		Pre	F1	Pre	F1	Pre	F1	Pre	F1	Pre	F1
1	*P. yunnanensis*	0.92	0.90	0.89	0.80	0.90	0.90	0.36	0.43	0.89	0.83
2	*P. armandii*	0.83	0.89	0.55	0.66	0.81	0.89	0.83	0.76	0.82	0.84
3	*P.taiwanensis*	0.77	0.77	0.45	0.28	0.84	0.84	0.23	0.18	0.71	0.70
4	*P. elliottii*	0.93	0.78	0.34	0.28	0.94	0.81	0.24	0.32	0.78	0.77
5	*P. bungeana*	0.97	0.96	0.71	0.74	0.89	0.93	0.92	0.93	0.85	0.89
6	*P. massoniana*	0.82	0.86	0.37	0.43	0.86	0.87	0.22	0.31	0.68	0.75
7	*P. thunbergii*	0.81	0.70	0.79	0.45	0.89	0.78	0.04	0.07	0.81	0.54
	Average	0.86	0.84	0.59	0.52	0.88	0.86	0.41	0.43	0.79	0.76
	Accuracy	0.84		0.54		0.86		0.46		0.76

**Table 5:** Precision and F1 scores of five pine nut classification models with normalized NIR spectral data.

Number	Type	DT		MLP		RF		SVM		NB	
		Pre	F1	Pre	F1	Pre	F1	Pre	F1	Pre	F1
1	*P. yunnanensis*	0.89	0.87	0.99	0.99	0.86	0.87	0.94	0.97	0.82	0.83
2	*P. armandii*	0.91	0.91	1.00	1.00	0.90	0.93	0.94	0.95	0.94	0.95
3	*P.taiwanensis*	0.85	0.85	0.98	0.99	0.93	0.90	0.90	0.88	0.64	0.69
4	*P. elliottii*	0.87	0.80	1.00	0.99	0.91	0.83	0.97	0.93	0.87	0.83
5	*P. bungeana*	0.96	0.93	0.98	0.95	0.90	0.93	0.95	0.95	0.89	0.88
6	*P. massoniana*	0.86	0.90	1.00	1.00	0.97	0.93	0.88	0.93	0.75	0.80
7	*P. thunbergii*	0.80	0.77	0.95	0.96	0.86	0.87	0.98	0.92	0.71	0.51
	Average	0.88	0.86	0.99	0.98	0.90	0.89	0.94	0.93	0.80	0.78
	Accuracy	0.87		0.99		0.89		0.94		0.80	

### Classification model based on image data

**Figure 2:**
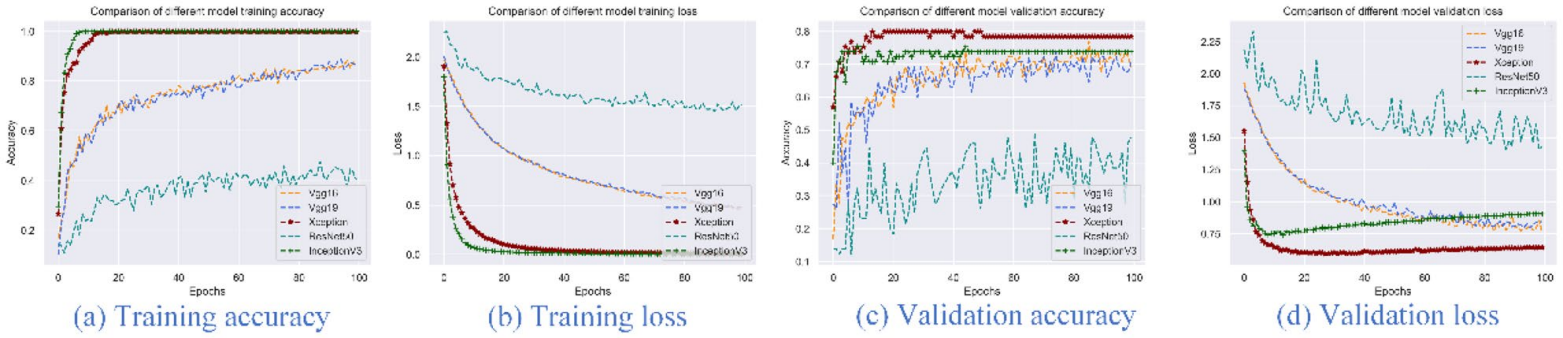
Accuracy and loss for five different models using image_clip data.

**Figure 3:**
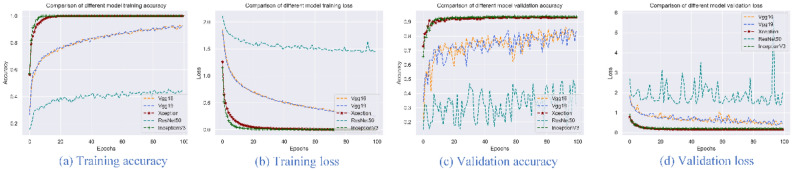
Accuracy and loss for five different models using image_trans data.

**Figure 4:**
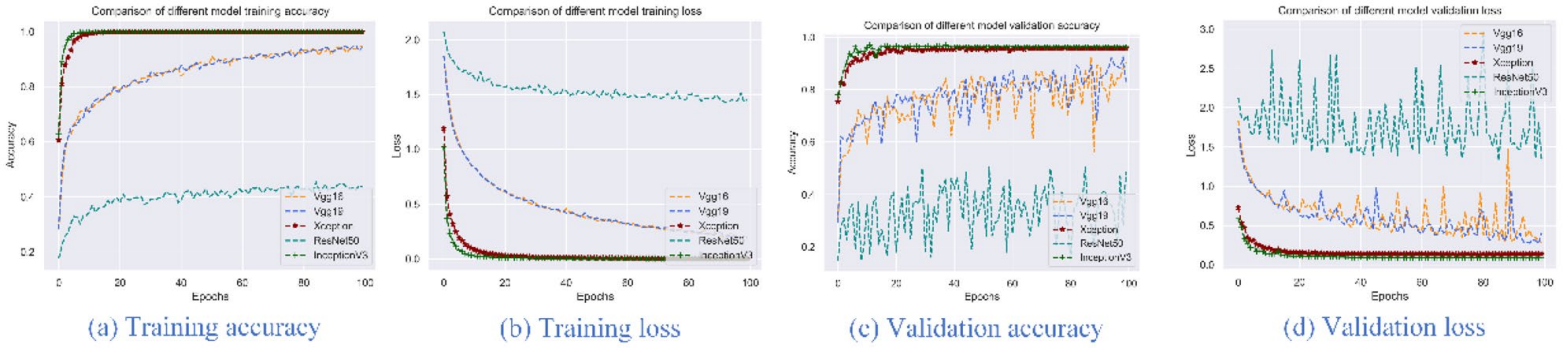
Accuracy and loss for five different models using image_gray data.

Three pre-processing methods were run for the datasets of image_clip (clipped images), image_trans (transformed images), and image_gray (grayscale transformed images). The image_clip data is used to explore the results of the deep learning model on the original data, image_trans and image_gray are obtained by extending the image_clip transformation. VGG16, VGG19, Xception, ResNet50 and InceptionV3 models were selected with the options of 100 epochs, and accuracy and loss were used as evaluation indicators. Figures 2, 3 and 4 present the accuracy and loss values of the five trained and verified models. From these figures, Xception and InceptionV3 have the best performance with the highest accuracy and lowest loss compared to the VGG16, VGG19 and ResNet50 models. Additionally, among the three pre-processing methods, image_trans outperforms image_gray and image_clip. Therefore, Xception and InceptionV3 models are best suited for image-based classification of pine nuts and images should be transformed but not set to grayscale (Table 6).

**Table 6:** Precision, F1 scores and accuracy of three pre-process methods.

Model	image_clip^a^			image_trans^b^			image_gray^c^		
	Pre	F1	Acc	Pre	F1	Acc	Pre	F1	Acc
VGG16	0.72	0.67	0.692	0.91	0.90	0.905	0.84	0.80	0.803
VGG19	0.72	0.68	0.708	0.87	0.82	0.826	0.86	0.83	0.836
Xception	0.81	0.79	0.785	0.96	0.96	0.957	0.93	0.93	0.931
ResNet50	0.59	0.44	0.477	0.52	0.46	0.485	0.45	0.25	0.311
InceptionV3	0.74	0.71	0.738	0.96	0.96	0.964	0.94	0.93	0.934

^a^image_clip, the clipped images.

^b^image_trans, the transformed images.

^c^image_gray, the transformed grayscale images.

## Discussion

Previous studies have shown that genus *Pinus* originated in the early Cretaceous (116–83 Mya) and diverged into two subgenera *Pinus* (*P. massoniana*, *P. thunbergii*, *P. yunnanensis*, *P. taiwanensis* and *P. massoniana*, etc.) and *Strobus* (*P. armandii* and *P. bungeana*, etc.)^2, 27^. During the long evolutionary history, it may have experienced many events such as plate movement, sea-land transition and climate changes^2, 28, 29^. The chemical composition of plant organs is the result of the interaction between plants and the environment in the long process of evolution^30–32^. Our results suggested that the species *P. armandii* and *P. bungeana* of subgenus *Strobus* have higher bands in regions 9,000–4,000 cm^−1^ (1,111–2,500 nm) than other five species of subgenus *Pinus* (Fig. 1). These bands were found to be associated with proteins, amino acids, moisture, lipids and carbohydrates in previous studies^20, 22^. Notably, our results also showed that three sensitive bands (1,147–1,154 nm, 1,907–1,927 nm, 2,227–2,254 nm) in these regions (1,111–2,500 nm) have great influence on the model accuracy based on sliding window method (Fig. 1). Different with subgenus *Pinus*, the species *P. armandii* and *P. bungeana* of subgenus *Strobus* were mainly distributed in Northern China (Table S1). The difference of some substances could be caused by certain geographical distribution and environmental conditions such as altitude, average annual temperature, soil characteristics, precipitation, and sunshine^22^. Compared with previous studies based on SVM, RF and PLS-DA methods in seed classification^12, 18^, our results showed that MLP model presented excellent performance, which could be explained that the collected NIR spectra were different in sensitivity to the model due to different chemical components.

We also found some morphological differences among two subgenera in pine nut images. The seeds of subgenus *Strobus* probably have a smoother shape and texture than subgenus *Pinus* (Fig. 7), which would be conducive to the feature extraction of machine learning model. Previous studies have shown that the PLS-DA and IPLS-DA models were achieved good results to recognize the multiple varieties of two species^22^. However, our results suggested that the InceptionV3 model performed best on the pine nut images of seven species with the fastest convergence speed and highest accuracy. The similar model was found to be successfully used to diagnosis of nutrient deficiencies in rice^33^ and classification of multiple weed species^34^. The different recognition accuracy of multiple models may be related to the morphological features (shape, color and texture) of nuts between datasets.

**Figure 5:**
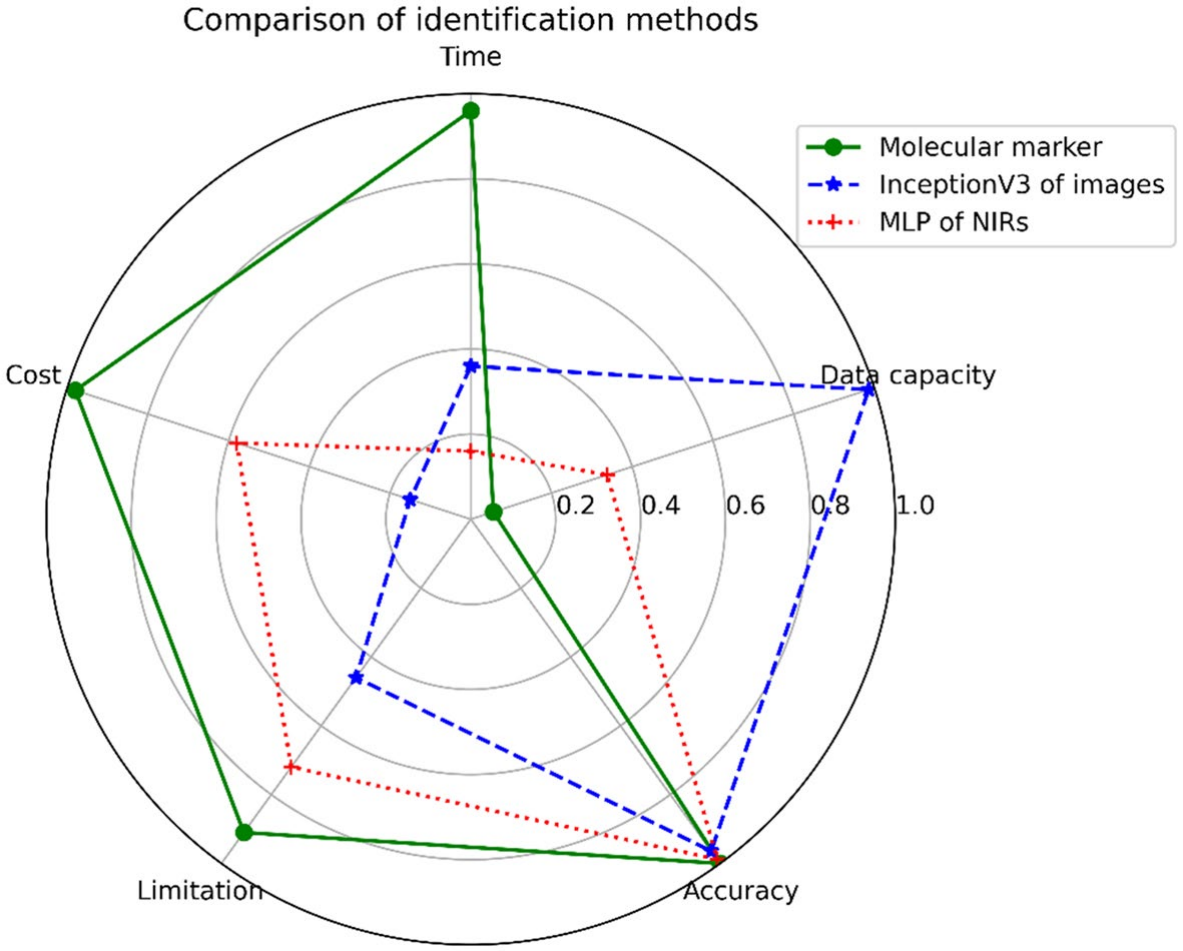
Radar chart of analytical costs, complexity and performance. Time: the time required for the analyses; Cost: the financial cost of completing the analyses; Limitation: the degree of limiting factors of experimental conditions; Data capacity: the amount of data obtained from the analyses; Accuracy: the accuracy of identification. The scale here represents value with 0 indicating the lowest value and 1.0 indicating the highest value.

There are different advantages in three recognition methods of molecular markers, NIR and images (Fig. 5). In terms of accuracy, molecular markers have higher recognition rates than NIR and images. However, molecular labeling takes a long time, as well as being limited by experimental equipment and public reference databases. In terms of cost, image analysis may be better, because it is convenient, fast and free from environmental constraints, but this method requires a large amount of images and has a lower recognition rate. In terms of performance, NIR spectroscopy may be better duo to its higher recognition rate and smaller amount of data generated, but it is costly and requires special devices. In the future, we would take advantage of the ensemble learning approach by merging multiple features of molecule, NIR and images for more species.

## Conclusions

Based on the present study findings, this study verifies the potential application of machine learning models based on NIR spectroscopy and images to recognition among different species of pine nuts. We collected seven species of pine nuts as the research object, constructed classification models based on NIR spectroscopy and image data. Compared with different models, MLP and InceptionV3 were proved to achieve better classification effect. At the same time, sensitive bands of NIR shows the correlation with some special molecular vibrations of functional groups. The results will provide solutions and scientific methods for the convenient, rapid and nondestructive classification of different species of pine nuts, and provide a new idea in the field of species classification, as well as a methodological and technical scheme for reference.

## Materials and methods

### Sample collection and pre-process

The academic permission to collect and study pine nuts was granted by the director of the Key Laboratory of Southwest Mountain Forest Resources Conservation and Utilization, Ministry of Education, Southwest Forestry University. The study met all relevant guidelines.

Used in the study of *P. bungeana* | Junying Jiao 01 |, *P. armandii* | Kunming Institute of Botany, Chinese Academy of Sciences, ZuoZh271 |, *P. yunnanensis* | Kunming Institute of Botany, Chinese Academy of Sciences, MY259|, *P. thunbergia* | Kunming Institute of Botany, Chinese Academy of Sciences, Lilan898 |, *P. massoniana* | Kunming Institute of Botany, Chinese Academy of Sciences, LWY2020020 |, *P. elliottii* | Junying Jiao 02 |and *P. taiwanensis* | Kunming Institute of Botany, Chinese Academy of Sciences, Jiangxc0597 | were prepared from the Kunming Institute of Botany, Chinese Academy of Sciences and Yunnan Forest seedling work station preparation plants. The pine nuts used in the study were formally identified by Junying Jiao, director of the Key Laboratory of Forest Resources Conservation and Utilization in Southwest Mountainous Region of the Ministry of Education, College of Forestry, Southwest Forestry University. *P. bungeana* and *P. elliottii* were registered and preserved in the herbarium of Southwest Forestry University, with code access number: 0000651 and 0,000,652. *P. armandi*, *P. yunnanensis*, *P. thunbergia*, *P. massonian*a and *P. taiwanensis* were registered and preserved in the Germplasm Bank of Kunming Institute of Botany, Chinese Academy of Sciences, with code access number: ZuoZh271, MY259, Lilan898, LWY2020020 and Jiangxc0597.

Approximately 1.5 kg of nuts from each species were selected and subjected to pre-treatment for image and NIR spectroscopy analyses. The seed surface was rinsed with distilled water, and defective nuts were removed. The cleaned pine nuts were then dried in an oven (Model DHG-9245A, Shanghai Hengke Instrument Co., Ltd., Shanghai, China) at 40 °C for 8 h. After pre-process, the nuts were randomly divided into 30 groups for subsequent acquisition of NIR spectra. One or two nuts from each group were photographed to obtain the origin images (Table 7).

**Table 7:** Pine nut images and NIR spectra.

Number	Species	NIRs^a^	image_clip	image_trans	image_gray
1	*Pinus bungeana*	30	42	210	210
2	*Pinus yunnanensis*	30	38	190	190
3	*Pinus thunbergii*	30	45	225	225
4	*Pinus armandii*	30	41	205	205
5	*Pinus massoniana*	30	52	260	260
6	*Pinus elliottii*	30	44	220	220
7	*Pinus taiwanensis*	30	41	205	205

^a^NIRs, the number of NIR spectra.

### Molecular markers

In order to identify pine nuts species, the primers of ITS2 and rbcL were designed based on the known sequences in a previous study^35^ (Table 8). Fragment genes were located and sequenced using an ABI 3730 sequencer. SeqMan tool was used to assemble the overlapping fragments.

**Table 8:** Primer reference for ITS2 and rbcL sequence.

Gene	Forward primer	Reverse primer
ITS2	5′-ATGCGATACTTGGTGTGAAT-3′	5′-GACGCTTCTCCAGACTACAAT-3′
rbcL	5′-ATGTCACCACAAACAGAAAC-3′	5′-TCGCATGTACCTGCAGTAGC-3′

### Spectral data acquisition and pre-process

The NIR spectra were acquired using the Antaris Fourier Transform NIR spectrometer (Thermo Fisher Scientific, Massachusetts, USA) equipped with an InGaAs detector with diffuse integrating sphere, a 7.78 cm quartz sampling cup and sample rotary table within the range of 12,800 to 3,800 cm^−1^ (781 nm-2632 nm) at a resolution of 8 cm^−1^. Samples were scanned 48 scanning times, and 2335 bands were obtained. The data were transformed using log(1/R) to represent absorbance.

The NIR spectra were normalized using a min–max normalization method to eliminate the adverse effects caused by outliers. The original data were normalized to the range of 0 and 1 using Eq. (1).

1 $${x}^{*}=\frac{x-\mathrm{min}\left(x\right)}{\mathrm{max}\left(x\right)-\mathrm{min}\left(x\right)}$$

where x represents absorbance values, min(x) and max(x)represent the lowest and absorbance highest absorbance values, respectively.

### Image acquisition

The pine nut images were captured using a LEICA EZ4 microscope with a white background and eightfold magnification through a Huawei Mate 30 mobile phone with a 40 MP ultrasensitive camera (wide angle, f/1.8) supporting auto focus and manual focus. The shooting angle was set to 90°, the height was 50 cm, and 52 images were taken for each species of pine nut.

### Image pre-process

During the image capturing process, irregularities arise. These include the size variation of pine nuts, inconsistent positions, and appearance of color, all of which will affect the recognition models and accuracy of classification. Thus, image pre-processing for standardization involved the following two steps:

(1) Edge detection and clipping

**Figure 6:**
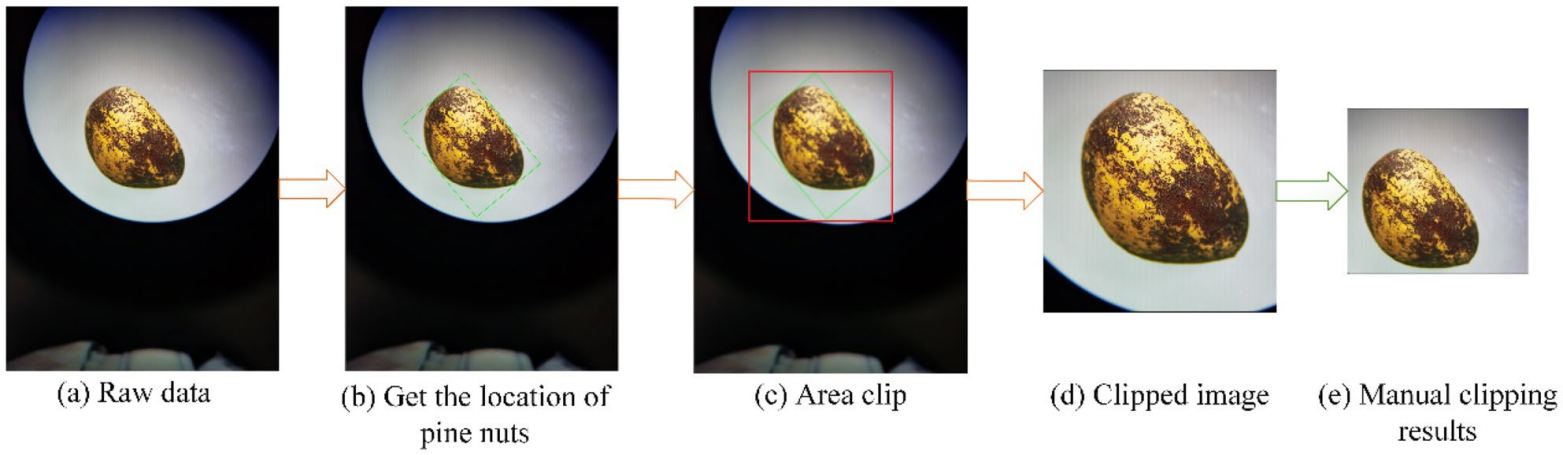
Sobel edge detection and clipping process.

The edge position of the pine nuts was detected with the Sobel method on the OpenCV platform. Once the top, bottom, left, and right vertices of the seed were de-fined, the image was cropped through a matrix frame connecting the four vertices (Fig. 6). In order to maintain a uniform image background (Fig. 6d), further manual cutting was sometimes necessary (Fig. 6e).

(2) Data augmentation and image grayscale

**Figure 7:**
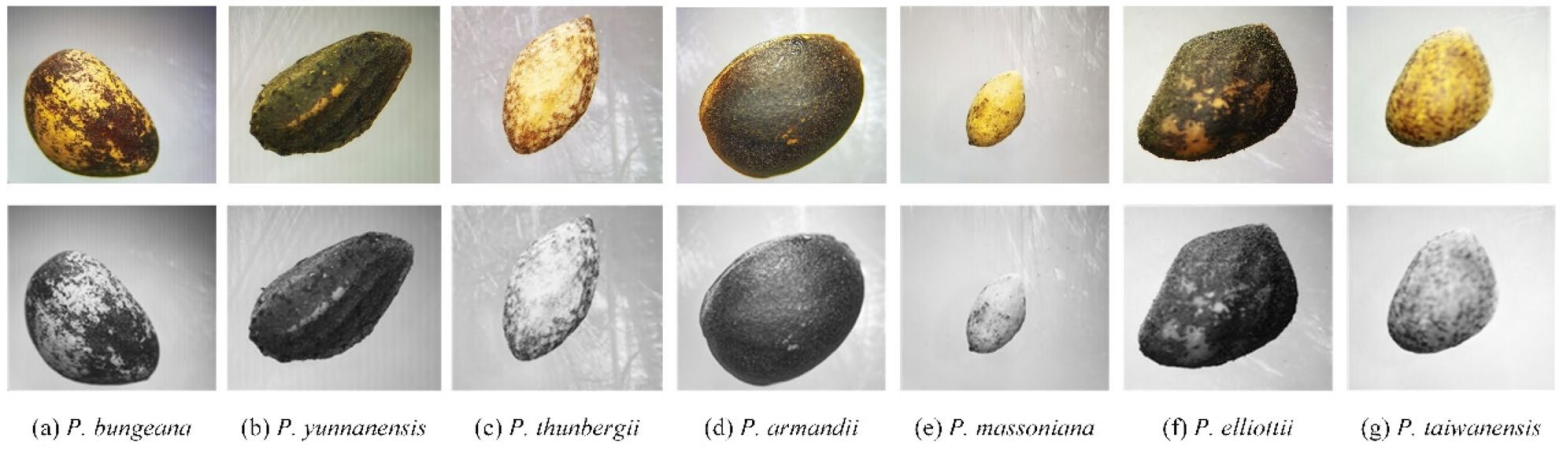
Results of image pre-processing for pine nuts of each species. Images have been clipped, flipped, resized and color transformed to grayscale.

The clipped images were oriented using the ‘flip’ and ‘resize’ functions in OpenCV. The formula (2) was used to transform these aligned images into grayscale images (Fig. 7). The OpenCV's color was used conversion function in this study: CV_BGR2GRAY to perform image grayscale processing.

2 $$\mathrm{Gray}=\mathrm{R}*0.299+\mathrm{G}*0.587+\mathrm{B}*0.114$$

### Structural design of pine nuts classification model

**Figure 8:**
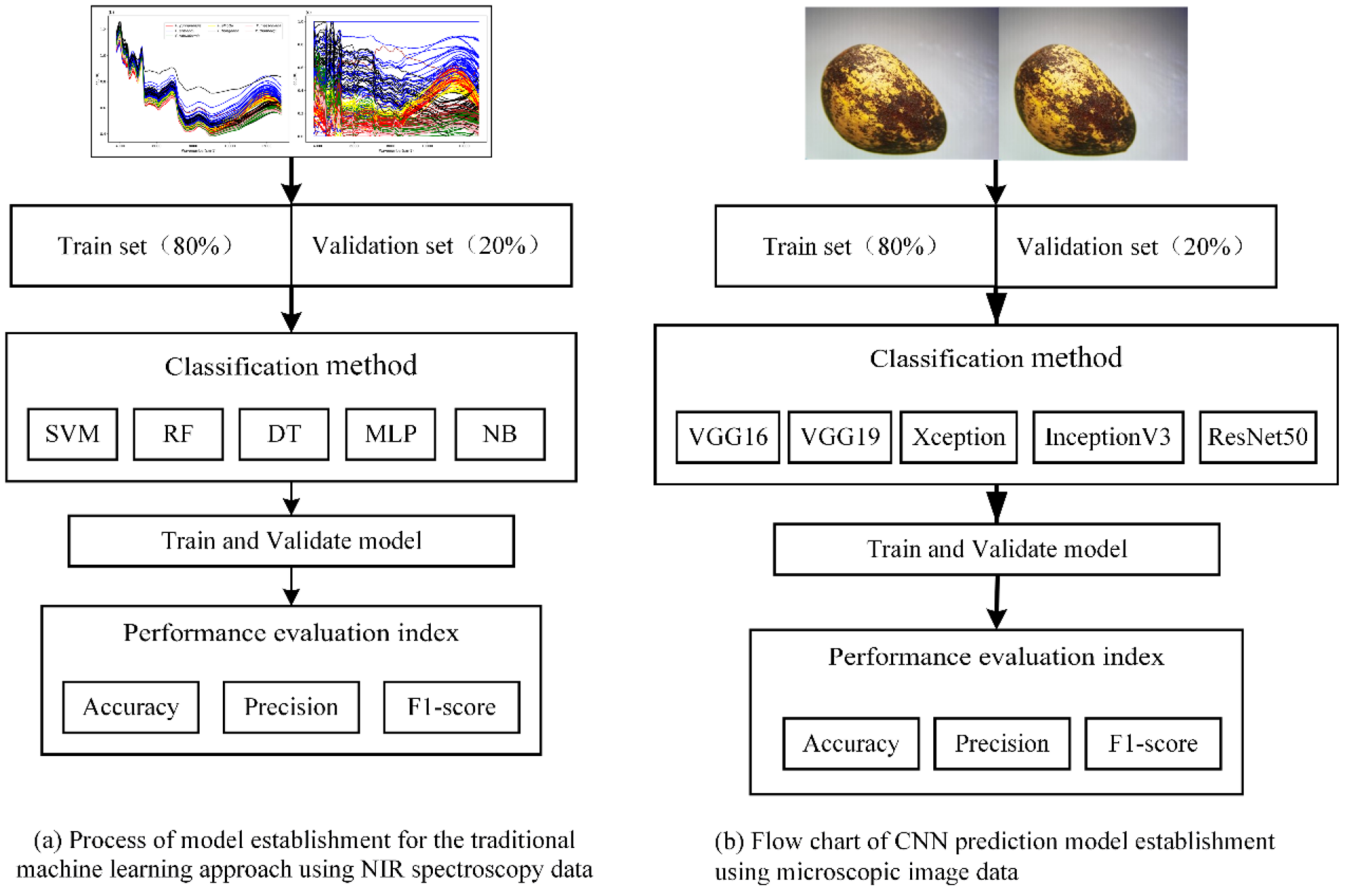
Experimental design process for image recognition and NIR spectroscopy. (**a**) Process of traditional machine learning classification model establishment using NIR spectroscopy data. (**b**) Process of deep learning classification model establishment using image data.

In order to further study the pine nut classification model, two experimental approaches were employed (Fig. 8). For the first approach involved traditional machine learning methods such as DT, RF, MLP, SVM and NB which were used to classify nuts based on the NIR spectroscopy. The classification model based on NIR spectra includes five steps (Fig. 8a). Data were first prepared and then divided into a training set and a validation set according to the ratio of 8:2. The DT, RF, MLP, SVM and NB learning methods were then used to establish classification models. Following training and validation, the accuracy (Acc), Pre, and F1 were selected as performance evaluation indicators of each classification model.

The second approach, five CNN models (VGG16, VGG19, Xception, InceptionV3 and ResNet50) were constructed and trained to classify the images of pine nuts (Fig. 8b). First, the original images in the dataset were of different sizes. Before the experiment, the original images were pre-processed and then cut into 224 × 224 sizes. Second, the pine nut images were divided into a training set and a validation set according to the ratio of 8:2. Then, the VGG16, VGG19, Xception, ResNet50 and InceptionV3 models were loaded on the experimental platform for training and validation. The epochs were set to 100 times, the Stochastic Gradient Descent (SGD) optimization method was adopted, and the initial learning rate was set to 0.005. The learning rate changes with training turns, with attenuation of 1e-6 per turned, and the momentum parameter was set to 0.9. The loss function was sparse_categorical_crossentropy, and the activation function was Rectified Linear Units (ReLU). Finally, the Acc, Pre, and F1 were selected for model evaluation.

These two experimental approaches were designed to compare and analyze the performance of different models to evaluate which one would best serve future research of pine nut classification. CNN models were built using the Python libraries Keras-nightly 2.6.0, TensorFlow-nightly-GPU 2.6.0, and Scikit-learn 0.24.2 run in Python v.3.7.

## Supplementary Information

Supplementary Table S1.

## Data Availability

The data and codes presented in this study are available in https://github.com/SWFU-JiangLiu/Recognition-of-pine-nuts.git. The GenBank accession numbers are OK271114-OK271122 and OK274058-OK274066.
